# The relational responding task: toward a new implicit measure of beliefs

**DOI:** 10.3389/fpsyg.2015.00319

**Published:** 2015-03-24

**Authors:** Jan De Houwer, Niclas Heider, Adriaan Spruyt, Arne Roets, Sean Hughes

**Affiliations:** ^1^Experimental-Clinical and Health Psychology, Ghent UniversityGhent, Belgium; ^2^Department of Developmental, Personality and Social Psychology, Ghent UniversityGhent, Belgium

**Keywords:** implicit measures, racism, relational responding

## Abstract

We introduce the Relational Responding Task (RRT) as a tool for capturing beliefs at the implicit level. Flemish participants were asked to respond *as if* they believed that Flemish people are more intelligent than immigrants (e.g., respond “true” to the statement “Flemish people are wiser than immigrants”) or to respond *as if* they believed that immigrants are more intelligent than Flemish people (e.g., respond “true” to the statement “Flemish people are dumber than immigrants”). The difference in performance between these two tasks correlated with ratings of the extent to which participants explicitly endorsed the belief that Flemish people are more intelligent than immigrants and with questionnaire measures of subtle and blatant racism. The current study provides a first step toward validating RRT effects as a viable measure of implicit beliefs.

Implicit measures have become an important part of the psychologist's toolbox (see Nosek et al., 2011; Gawronski and De Houwer, 2014, for recent reviews). Although opinions differ on what it means to say that a measure is implicit, we favor the view that implicit measures are measurement outcomes that capture the to-be-measured construct (e.g., attitudes, stereotypes, evaluation) under conditions of automaticity (e.g., even when participants have little time, are engaged in multiple tasks, are not aware of what is being measured, or do not have the intention to express the construct that is being measured; see De Houwer et al., 2009; De Houwer and Moors, 2012). In most implicit measurement tasks, participants are asked to respond as quickly as possible to stimuli that appear on a computer screen. For instance, in an Implicit Association Test (IAT) designed to assess implicit self-esteem, positive words, negative words, stimuli related to the self (e.g., the first name of the participant), and stimuli related to other people (e.g., the first name of another participant) appear one by one on a computer screen. In a first critical block of trials, participants are asked to press one key as quickly as possible whenever they see a positive word or a self-related stimulus and a second key whenever they see a negative word or an other-related stimulus. In a second critical block, positive words and other-related stimuli are assigned to the first key whereas negative words and self-related items are assigned to the second key. Implicit self-esteem is inferred from the difference in performance in the two critical blocks. For instance, participants who perform better in the first relative to the second block are assumed to have more positive self-esteem than participants who perform better in the second relative to the first block (e.g., Greenwald and Farnham, 2000).

A core property of most implicit measures is that they were designed to capture associations between concepts while ignoring the way in which those concepts are related (see Hughes et al., 2012, for a detailed overview). For instance, the propositional beliefs “I am good” and “I want to be good” both involve a relation between “I” and “good” but differ with regard to the type of relation. Even though these beliefs are fundamentally different and might coincide with entirely different behaviors, an IAT cannot differentiate between them. The Implicit Relational Assessment Procedure (IRAP), on the other hand, does allow one to differentiate at the implicit level between beliefs that differ only with regard to the relational component (for an overview, see Barnes-Holmes et al., 2010)^1^.

Consider a version of the IRAP designed to capture the belief “I am good.” On each trial of this task, either “I am” or “I am not” appears at the top of a computer screen along with a positive or negative adjective in the middle of the screen (e.g., “good” or “bad”). In this way, the task is comprised of four different types of trials (*I am + positive; I am + negative; I am not + positive; I am not +negative*). As with the IAT, there are two different types of critical blocks. In one block of trials, participants are trained to select the response “correct” on trials that are in line with the belief “I am good” (i.e., *I am + positive*; *I am not + negative*) and to respond “false” on trials that contradict this belief (i.e., *I am not + positive* and *I am + negative*). In the second type of blocks, responses need to be in line with the belief “I am bad” (i.e., respond “correct” on *I am not + positive* and *I am + negative* trials and respond “false” on *I am + positive* and *I am not + negative* trials). The better someone performs on the first type of blocks relative to the second type of blocks, the stronger that person is assumed to hold the belief “I am good” (relative to “I am bad”)^2^.

Importantly, this version of the IRAP can easily be redesigned to capture the belief “I want to be good.” The only necessary change is to replace the stimuli “I am” and “I am not” with the stimuli “I want to be” and “I do not want to be,” respectively. Once this change is in place, participants can be trained to select the response “correct” on *I want to be + positive* and *I do not want to be + negative* trials and to select the response “false” on *I want to be + negative* and *I do not want to be + positive* trials. During a second type of blocks, they are required to select “correct” on *I want to be + negative* and *I do not want to be + positive* trials and to select “false” for *I want to be + positive* and *I do not want to be + negative* trials. Comparing performance across these different types of blocks provides an index of the belief “I want to be good” (relative to “I want to be bad”).

The significance of distinguishing between different beliefs at the implicit level was recently demonstrated in a study by Remue et al. (2013; see also Remue et al., 2014). Their research was triggered by the observation that implicit self-esteem as indexed by IAT scores is as positive in dysphoric students and acutely depressed patients as it is in non-dysphoric students or non-depressed control participants (e.g., De Raedt et al., 2006). Remue and colleagues tested the idea that standard implicit measures of self-esteem might reflect different types of beliefs in different participants. For instance, in non-dysphoric students, positive self-esteem IAT scores might reflect the belief “I am good” (i.e., actual self-esteem) whereas the same score might reflect the belief “I want to be good” (i.e., ideal self-esteem) in dysphoric students. In line with this hypothesis, they observed that dysphoric students had lower scores on an IRAP designed to capture the belief “I am good” than on an IRAP designed to capture the belief “I want to be good” whereas non-dysphoric students showed the reversed pattern.

The IRAP has been used successfully in a wide variety of contexts (see Barnes-Holmes et al., 2010; Hughes and Barnes-Holmes, 2013, for reviews). Still it would be good to have alternative measures of implicit beliefs, if only to assess and control for method specific variance. Moreover, the IRAP is often quite difficult for participants to complete. Across many studies, a substantial part of the (university student) sample fails to complete an IRAP (e.g., more than 20% of the students in the study of Remue et al., 2013; see Hughes and Barnes-Holmes, 2013, Table 1, for an overview). The difficulty of the IRAP originates—at least in part—from the fact that the assignment of keyboard keys to the “correct” and “false” response option typically varies from trial to trial (e.g., press d for “correct” and k for “false” on trial n but press d for “false” and k for “correct” on trial n + 1). The assignment of responses to different keys is varied in order to avoid a potential confound between the function of the response (indicate “correct” or “false”) and its physical location (e.g., the “d” or “k” keys). This confound could alter the encoding of the responses by allowing participants to select a response on the basis of its spatial properties (e.g., “if *I am + positive*, then press key d”) rather than its meaning (e.g., “if *I am + positive*, then press correct”; but see (Campbell et al., 2011), for data showing that keeping the assignment of responses fixed does not necessarily reduce IRAP effect sizes). Although it might be possible to avoid a high drop-out rate by increasing prior exposure to the task or changing properties of the procedure (see Vahey et al., 2010; Hughes and Barnes-Holmes, 2013, for a discussion), the difficulty in completing an IRAP does constrain its utility, especially when using it via online applications (in which case extensive training is often not feasible) or with certain populations (e.g., clinical patients with limited attentional or intellectual capacities).

In the present paper, we offer the Relational Responding Task (RRT) as a novel measurement procedure that, like the IRAP, is designed to capture specific beliefs at the implicit level. It retains an essential ingredient of the IRAP—namely—the requirement for participants to respond in-line with specific beliefs (e.g., “I am good” or “I want to be good”). Unlike most instantiations of the IRAP, the RRT involves the presentation of full statements in the middle of the computer screen (e.g., “I like myself”) and participants are explicitly instructed to act “as if” they agree with certain statements and disagree with others^3^. For instance, in a first block, participants might be asked to respond as if they believe that they are good by selecting “true” when presented with statements that imply positive views of the self (e.g., “I like myself”) and by selecting “not true” when presented with statements that imply negative views of the self (e.g., “I dislike myself”). In a second block, they would respond as if they believe that they are bad by selecting “true” when presented with negative self-related statements and “false” when presented with positive-self related statements. The difference in performance between these two types of blocks is assumed to provide a measure of the extent to which participants believe that they are good.

In addition to differences in the type of stimuli used and the nature of the instructions, the IRAP and RRT also differ in their structural properties. In the RRT, the physical properties of the correct and false response remain constant throughout the task. On all trials, responding “true” is realized by pressing a first key whereas responding “not true” is done by pressing a second key. Recoding of the responses (e.g., in terms of physical location) is discouraged, however, by including inducer trials on which stimuli are presented that refer to the concepts “true” or “not true” (e.g., words such as “correct” and “wrong”). Participants are instructed to press one key for inducer stimuli that refer to “true” and to press a second key for inducer stimuli that refer to “false.” Similar inducer trials have been used successfully in other implicit measurement procedures as a means to discourage recoding of the responses (e.g., Extrinsic Affective Simon Task; De Houwer, 2003).

Interestingly, the inclusion of inducer trials results in the RRT having a task structure that closely resembles the IAT. More specifically, in both the RRT and the IAT, four categories of stimuli are assigned to two responses in a way that varies across blocks. As a result, it is likely that the difficulty of performing an RRT will be similar to the difficulty of performing an IAT. Given that (a) attrition rates in IAT studies are typically low, that (b) IATs have already been successfully administrated via the world wide web on a large scale, and that (c) IATs have been used successfully with a variety of populations (e.g., Nosek et al., 2007, for a review), we believe that the convenience of use of the RRT will be high, and in several respects, superior to that of the IRAP (at least as currently instantiated).

Despite their structural similarity, it is important to realize that the IAT is fundamentally different from the RRT (and IRAP) in several ways. Most importantly, a typical IAT does not require participants to relate the different categories that are used in the IAT. In a self-esteem IAT, for example, participants can select the correct response merely by identifying the individual category that the presented stimulus is a member of (e.g., the word “me” is a member of the category of stimuli labeled “self”). In the RRT (and the IRAP), on the other hand, participants can only select the correct response based on the way that different stimuli are related to one another. For instance, in an RRT designed to assess the belief “I am good,” a correct response does not simply depend on the presence of specific stimuli (e.g., “I,” “am,” or “good”) but on the relation *between* different stimuli (e.g., the statement “I am good”). Moreover, the instruction to “act as if” statements are true or false encourages participants to relate the presented statement with the categories “true” and “not true.” That is, participants need to respond to a statement as being either true or false based on the rule that is specified for each block of trials. In sum, the RRT requires participants to respond in a complex relational manner (hence the name *relational responding* task). It is precisely this feature of the RRT that endows it with the potential to capture individual beliefs that differ with regard to the relational component (e.g., “I am good” vs. “I want to be good”).

In the current paper, we set out to validate empirically the RRT effect as an implicit measure of beliefs. Rather than immediately testing the potential of the RRT to capture differences between beliefs that vary only in their relational component (e.g., by comparing an RRT designed to capture the belief “I am good” with an RRT that captures the belief “I want to be good”), we first conducted a study that focused on a single belief and examined whether an RRT measure of that belief correlates with criterion variables (see De Houwer et al., 2009, for more information on how to validate implicit measures). More specifically, we used an RRT that was designed to capture the extent to which Flemish participants hold the belief that Flemish people are more intelligent than immigrants. The inducer stimuli were synonyms of the concepts “true” or “false” that had to be categorized as either “true” by pressing one key or as “false” by pressing another key. Target trials involved the presentation of statements that were either in line with the belief that Flemish people are more intelligent that immigrants (e.g., “Flemish people are smarter than immigrants,” “Immigrants are dumber than Flemish people”) or in line with the belief that immigrants are more intelligent than Flemish people (e.g. “Flemish people are dumber than immigrants,” “Immigrants are smarter than Flemish people”).

In addition to practice blocks that contained either inducer trials or target trials only, the RRT also included two types of test blocks that contained both inducer and target trials. During the first type of test block, participants were asked to respond to the target statements as if they believed that Flemish people are more intelligent than immigrants (i.e., to respond “true” to statements such as “Flemish people are smarter than immigrants” and to respond “not true” to statements such as “Flemish people are dumber than immigrants”; pro-Flemish block). In the second type of test block, participants were asked to respond as if they believed that immigrants are more intelligent than Flemish people (i.e., to respond “not true” to statements like “Flemish people are smarter than immigrants” and to respond “true” to statements like “Flemish people are dumber than immigrants”; pro-immigrant block). The difference in performance across these two types of blocks is assumed to assess the belief that Flemish people are more intelligent than immigrants.

In order to validate the RRT scores, we also asked participants to express on a rating scale the extent to which they believe that Flemish people are more or less intelligent than immigrants. Because implicit and explicit measures of the same construct typically converge to some extent (Nosek, 2007), we expected that the RRT and rating measure would be correlated. Participants also completed questionnaires designed to capture subtle, modern, and blatant forms of racism. Blatant racism is considered to be a hot and direct form of racial prejudice, often referring to beliefs about the (genetic) inferiority of the racial out-group. It is typically associated with a strong opposition to any intimate contact with the out-group. Subtle racism, on the other hand, is a more cool and covert expression of racial prejudice, including the idea that out-groups are poorly adapted to the ingroup's traditional values, an exaggeration of cultural differences between the in-group and the out-group, and the absence of positive emotions (rather than the presence of negative emotions) toward the out-group (see Pettigrew and Meertens, 1995). Similarly, modern racism also refers to a more covert form of prejudice (as opposed to the blatant, old-fashioned forms) but is distinct in that it taps into beliefs that racial discrimination is no longer a problem (or even does not exist anymore) and that out-groups (i.e., Black people in the original work) have become too demanding and are pushing for unfair advantages (McConahay, 1986). If a Flemish person believes that Flemish people are more intelligent than immigrants, this belief could be regarded as an instance of racial prejudice, especially blatant/old-fashioned racism (e.g., McConahay, 1986; Bobo and Kluegel, 1993). We therefore expected that RRT scores would also correlate with scores on these racism questionnaires, in particular blatant racism.

Finally, please note that we did not make predictions about the direction or magnitude of the overall RRT score. First, the overall RRT score in our study would probably be biased as the result of order effects. We fixed the order of the blocks (i.e., all participants started with the pro-Flemish block) because we were interested primarily in interindividual differences in RRT scores, that is, we wanted to correlate RRT scores with other measures. Counterbalancing block order is known to increase error variance (i.e., differences in scores between participants might reflect not only differences in the to-be-measured attribute but also differences in block order) and thus to lower correlations (see Perugini and Banse, 2007, for a discussion and the recommendation to fix block order). Because there are few studies about the effect of block order on the validity of implicit measures (e.g., studies examining whether correlations with validity criteria are stronger when starting with an attitude-inconsistent vs. an attitude-consistent block), we did not have strong reasons to select one of the two block orders but more or less randomly decided to always start with the pro-Flemish block. More importantly, fixing the order of blocks complicates the interpretation of overall scores because those scores could be influenced by the order in which the blocks are completed. For instance, practice with the stimulus-response mappings in the first critical block might slow down responding in the second critical block during which those mappings are reversed. On the other hand, performance in the second critical block might be facilitated because of practice effects (e.g., faster responding because items are more familiar and thus easier to process). When block order is fixed, it is impossible to determine to what extent the overall score reflects the reversal of stimulus-response mappings, practice effects, or the properties of the to-be-measured attribute. Hence, it is best to refrain from interpreting the overall score when block order is fixed. A second reason for refraining from an interpretation of the overall RRT score is that, even without biases due to fixed block order, it is difficult to determine the correct interpretation of the zero point on psychological measures (Blanton and Jaccard, 2006).

## Methods

### Participants

Forty-nine students at Ghent University (*M*_age_ = 23 years, *SD*_age_= 4; five men) participated in exchange for €5. Ghent University is situated in Flanders, which is the northern region of Belgium. These participants were classified as being Flemish because they indicated that Dutch (which is the dominant language in Flanders) was their native language and that both their parents were Flemish. The data of six other participants who told the experimenter that they had at least one parent of non-Belgian nationality or said that Dutch was not their (only) native language, were excluded from the analysis. All participants reported normal or corrected-to-normal vision and provided their informed consent before participating.

### Materials

#### RRT

Ten words and 20 statements were used as stimuli. Five words were related to “true” (the Dutch words “goed,” “juist,” “correct,” “exact,” and “in orde”) and five words were related to “false” (the Dutch words “mis,” “onjuist,” “incorrect,” “verkeerd,” and “fout”). These words were presented during the inducer trials. Each of the 20 target statements (see Supplementary Material) related *Flemish people* and *immigrants* to one another in terms of their intelligence level, using five synonyms for intelligent (e.g., smarter) and five synonyms to denote a lack of intelligence (e.g., dumber). Ten statements implied that Flemish people are more intelligent than immigrants (e.g., “Flemish people are smarter than immigrants,” “Immigrants are less clever than Flemish people”) while another 10 statements implied that immigrants are more intelligent than Flemish people (e.g., “Immigrants are wiser than Flemish people,” “Flemish people are less wise than immigrants”). All statements were presented in bold Verdana font, size 28. Inducer stimuli were presented in orange, whereas target statements were presented in blue throughout the task. The experiment was conducted using a 17 inch LCD screen (60 Hz, 1440 × 900 pixels). The RRT program was written in Affect 4.0 (Spruyt et al., 2010), a copy of which can be downloaded at http://www.liplab.ugent.be/.

#### Questionnaires

Participants completed a 12-item subtle racism scale and an 8-item blatant racism scale (Pettigrew and Meertens, 1995; adapted by Van Hiel and Mervielde, 2005), along with a 10-item modern racism scale (McConahay, 1986; translated by Dhont et al., 2010). Virtually all questions referred to immigrants in general, with the exception of a few questions that referred to Turks and Moroccans, the two largest groups of immigrants in Belgium from outside of the European Union. Also, a few items stated that immigrants differ in their religious beliefs or culture from Belgians, implying that immigrants do not include foreigners from other western European countries. Examples are “Immigrants should be wise enough not to impose themselves at places where they know beforehand that they would be discriminated” (subtle racism scale), “Discrimination against immigrants no longer is problem in Belgium” (modern racism scale), and “Immigrants are a threat to the employment of Belgians” (blatant racism). Note that some questions referred to Belgian people as the in-group whereas the RRT referred to Flemish people as the in-group. If anything, this difference would reduce the magnitude of the correlations between the racisms measures and the RRT and thus work against our hypotheses. However, we did not expect a strong negative impact of this divergence because our participants are both Belgian and Flemish. Hence, both labels describe the in-group correctly as opposed to the out-group of immigrants.

All questions were rated on 7-point Likert scales ranging from 1 (“completely disagree”) to 7 (“completely agree”). In addition, participants were asked to provide their opinion on the relative intelligence of Flemish people and immigrants using a scale that ranged from -10 (immigrants are more intelligent than Flemish people) to +10 (Flemish people are more intelligent than immigrants), with 0 indicating that the two groups do not differ in their intelligence.

### Procedure

Participants were tested individually in a dimly lit room. All participants first completed the RRT. They were instructed to categorize words and statements presented on the screen as either “true” or “not true” by pressing the left or right control-keys of the keyboard, respectively. In the first block (40 trials), each of the 10 inducer words was presented 4 times as practice. Participants were asked to categorize the words as synonyms of “true” (press right control key) and “not true” (press left control key). In the second block of 40 trials, the 20 target statements were presented twice. Participants were asked to respond to the statements in line with the rule that Flemish people are more intelligent than immigrants (and immigrants are therefore less intelligent than Flemish people). The third block consisted of two consecutive repetitions of 40 trials, in which the 10 inducer stimuli were presented twice and the 20 target statements were presented once, leading to a total of 80 trials. Participants were asked to respond in accordance with the rules practiced in the two preceding blocks. Block 4 was identical to the second block except for a reversal of the rule for responding. That is, participants were asked to respond as if immigrants are more intelligent than Flemish (and Flemish are therefore less intelligent than immigrants). The fifth and final block was identical to the third, but participants were asked to respond to target statements in accordance with the response rule learned in block four.

The order of the trials was random except for the restriction that the same statement or word could not be presented on two consecutive trials. Each trial started with the presentation of a word or statement in the middle of the screen. It remained there until a response was registered. Incorrect responses were followed by the presentation of a red cross which remained on screen until participants gave the appropriate response. The following trial started 750 ms after a correct response was emitted. Once they completed the RRT, participants rated the target belief after which they completed the three questionnaires (subtle, modern, and blatant racism scale, in that order). They then were debriefed and paid.

## Results

### RRT data

Given that inducer trials were included merely to prevent response recoding, only data from the target trials of the mixed blocks were analyzed. Analyses that included also the data of the inducer trials led to the same conclusions. The data from two participants were excluded from the analyses because their percentage of errors was more than 2.5 standard deviations above the mean percentage of errors of the total group. Reaction times were defined as the time in milliseconds between the onset of presentation of the statement and the registration of the correct response. These reaction times were transformed into D_RRT_ scores using the same improved D-algorithm (D1) that Greenwald et al. (2003) developed for the IAT. The D1 score was chosen because we recorded reaction times until the correct response was emitted, thus removing the need for algorithms that add extra penalty time to reaction times on trials with an incorrect response. Analyses using the D4 (also known as D600) scoring algorithm, led to similar conclusions. D_RRT_ effects were scored so that positive values indicated faster responses in the pro-Flemish block (i.e., act as if Flemish people are more intelligent than immigrants) relative the pro-immigrant block of the RRT (i.e., act as if immigrants are more intelligent than Flemish people) while negative values indicated the opposite. The D_RRT_ score ranged from −0.79 to 0.56, with a mean D_RRT_ score of *M* = −0.01 (*SD* = 0.34). The mean D_RRT_ score did not differ significantly from zero, *t* < 1. During the mixed blocks, participants on average took 1551 ms (*SD* = 373.93) to respond to the target statements and 672 ms (*SD* = 88.58) to respond to the inducer words. In these blocks, they made on average 11.1% of errors (*SD* = 0.06) on trials with target statements and 5.3% of errors (*SD* = 0.04) on trials with inducer words.

### Correlation analyses

Table 1 provides an overview of all pairwise correlations between the D_RRT_ score, the explicit rating, and the three questionnaires. Most importantly, the D_RRT_ score correlated significantly with the explicit judgments of the relative intelligence of Flemish and immigrants, *r* = 0.43, *t*_(44)_ = 3.17, *p* = 0.003, with the scores on the subtle racism scale, *r* = 0.36, *t*_(45)_ = 2.59, *p* = 0.013, and with the scores on the blatant racism scale, *r* = 0.34, *t*_(45)_ = 2.39, *p* = 0.020. The correlations between the D_RRT_ score and those obtained from the modern racism scale did not reach conventional levels of significance, *r* = 0.20, *t*_(45)_ = 1.39, *p* = 0.171.

**Table 1:** **Correlations between measures**.

		**1**	**2**	**3**	**4**	**5**
1	D_RRT_	–	0.43^*^	0.36^*^	0.20	0.34^*^
2	Rating		–	0.40^*^	0.31^*^	0.70^*^
3	Subtle racism			–	0.74^*^	0.80^*^
4	Modern racism				–	0.67^*^
5	Blatant racism					–

* *p < 0.05*.

Odd-even split half reliability of the RRT score, using Spearman-Brown correction, was *Rsb* = 0.64. For the questionnaire of subtle, modern, and blatant racism, Chronbach's alpha was 0.85, 0.83, and 0.86, respectively. The mean value on those questionnaires was 4.26 (*SD* = 0.86), 3.55 (*SD* = 0.87), and 2.61 (*SD* = 1.05), respectively. Finally, the mean score on the explicit rating was 1.28 (*SD* = 1.05) which differed from zero, *t*_(45)_ = 4.41, *p* < 0.001.

## Discussion

The current study sought to provide a first step toward the validation of the RRT effect as an implicit measure of beliefs. In line with the hypothesis that the RRT scores capture the (prejudiced) belief that Flemish people are more intelligent than immigrants, we observed a significant correlation between RRT scores and explicit ratings of how much participants endorsed this belief. RRT scores also correlated positively with questionnaires of subtle and blatant (but not modern) racism. This pattern of correlations supports the claim that the RRT effects captured the to-be-measured belief and hence provides preliminary evidence for the idea that the RRT can provide a general tool for assessing beliefs. In addition, the RRT seems to be more user friendly than the IRAP. First, the attrition rate in our study was low. All participants successfully completed the RRT. We did discard the data of two participants because their percentage of errors was more than 2.5 standard deviations above the mean percentage of errors of the total group. However, this still implies an attrition rate of less than 5% (i.e., 4.08%) whereas attrition rates of 20% or more are common in IRAP studies. Moreover, participants needed only about 10 min to complete the RRT whereas it often takes 20 min or more to complete the various stages of the IRAP.

We not only put forward the claim that RRT scores capture beliefs but also that they provide an *implicit* measure of those beliefs. It has been argued that a measure qualifies as implicit if it functions as a valid measure even under conditions of automaticity (De Houwer et al., 2009; De Houwer and Moors, 2012). Based on the structural features of the RRT, one could argue that RRT scores provide an implicit measure in the sense that they capture beliefs even though participants are asked to respond quickly. Speed is of course a continuous variable, which implies that measures differ in the degree to which they are implicit along this criterion. In our study, for example, participants required an average of 1551 ms to respond to the target statements and 672 ms to respond to inducer words. Although 1551 ms might seem long relative to the reaction times observed in most other implicit measurement tasks, the target statements used in our study are much more complex than the single words or pictures that are usually presented in other implicit measurement tasks. Hence, it seems likely that participants did respond very quickly once they processed the meaning of the statements. Consistent with this conclusion, reaction times on inducer trials (during which participants reacted to individual words) were much shorter and comparable to those seen in other implicit measurement tasks. Regardless of how RRT scores relate to other implicit measures in this regard, it seems safe to argue that RRT scores are more implicit in terms of speed than scores obtained from traditional self-report procedures (e.g., ratings, questionnaires) that allow for ample time to reflect and respond to questions.

Are RRT measures implicit in the sense of unintentional? Unlike explicit measures such as the rating measures used in our study, participants are not asked to express their beliefs during an RRT. Instead, they are simply asked to *act as if* they endorse a certain belief. On the one hand, when asked to act in ways that contradict their beliefs, it is unlikely that participants will express those beliefs in an intentional manner because this would lead to incorrect responses. On the other hand, when asked to respond in line with beliefs that they do endorse, it is possible (but not necessarily the case) that participants do express their beliefs in an intentional manner rather than act as if they endorse those beliefs. Hence, at present, it is difficult to make strong claims about whether RRT measures are implicit in the sense of valid in the absence of the intention to express a belief. Future work could examine this issue further by determining the strategies that participants use while completing an RRT. Such work could also examine if the RRT meets other conditions of automaticity, such as controllability.

What would it mean to say that RRT scores capture beliefs at the implicit level? One way to conceptualize this statement is in terms of the implicit endorsement of propositions. At first sight, the idea that propositions can be endorsed implicitly might seem self-contradictory in light of cognitive theories that postulate that all implicit processing relies on associative representations (e.g., Rydell and McConnell, 2006). Unlike propositions, associations do not have a truth value: an association does not imply a statement about the world and is therefore neither true nor untrue. Hence, associations as such cannot be endorsed (i.e., be evaluated as true).^4^ Together, these two assumptions (all implicit processing relies on associations; associations cannot be endorsed) imply that endorsement can never be implicit.

More recently, however, cognitive researchers have raised the possibility that implicit processing can involve propositional representations (Hughes et al., 2011, 2012; De Houwer, 2014). Indeed, a rapidly growing body of evidence supports the conclusion that propositions can be both formed and activated under the various conditions of automaticity (see De Houwer, 2014, for a review). At the same time, Shidlovski et al. (2014), recently advanced the concept of “implicit truth” which they define as the automatic endorsement of propositions. Across several studies, the authors showed that indices of implicit truth can be dissociated from the explicit endorsement of truth. From this perspective, scores on the RRT (and IRAP) do have a unique and potentially crucial role to fulfill in psychological research as measures of the implicit endorsement of propositions.

One could argue, however, that the IRAP and RRT are not alone in their ability to capture beliefs at the implicit level. For instance, several variants of the IAT use categories that specify relational information. Consider the so-called personalized IAT (pIAT) in which participants classify (attribute) stimuli as instances of the categories “I like” or “I dislike” rather than the categories “good” or “bad” (Olson and Fazio, 2004; see Dewitte and De Houwer, 2008; Yoshida et al., 2012, for related propositionalized variants of the IAT). For instance, in a smoking pIAT (e.g., De Houwer et al., 2006), participants see words that refer to things they like (e.g., flowers), words that refer to things they dislike (e.g., cockroaches), pictures related to smoking (e.g., a lighter), and pictures unrelated to smoking (e.g., a pencil). One could argue that, in this case, the categorization of words is no longer based on category membership as such (i.e., good, bad, or I) but rather on the basis of the relation between different concepts (“I” in combination with “like” or “dislike”).

Nevertheless, while performance in the pIAT might be relationally more complex than performance on traditional IATs, the pIAT remains fundamentally different from the RRT (and IRAP). Like other IATs, the pIAT does not require participants to relate the different components of a to-be-measured belief. For instance, in a smoking pIAT, response selection is determined on the basis of individual elements of the beliefs “I like smoking” or “I dislike smoking” (i.e., the individual categories “I like,” “I dislike,” “smoking,” or “non-smoking”). In the RRT, on the other hand, response selection is instructed in terms of the full combination of these elements (i.e., “I like smoking” or “I dislike smoking”). Moreover, as we pointed out in the introduction, in the RRT but not in IATs, participants are instructed to respond on the basis of whether a statement is to be considered as true or false in a specific block of trials.

The autobiographical IAT (aIAT; see Verschuere et al., 2015, for a review) is another variant of the IAT that might allow for the measurement of beliefs and seems to bear even greater similarity to the RRT at the structural level than other IATs. In an aIAT, participants encounter generic statements that are true (e.g., “I am a human being”) or false (e.g., “I am a cow”) for all participants. They are required to classify these statements as either “true” or “false” by pressing one of two keys. Participants also see statements that describe a particular event (Event A) that the participant has witnessed (autobiographical items; e.g., statements related to a crime) or a second event (Event B) that the participant has not witnessed (control items). In one of the critical test blocks, participants press the same key for true items and items referring to Event A and a second key for false items and items referring to Event B. In the second critical test block, the stimulus-response assignments are reversed (press the first key for true items and Event B items; press the second key for false items and Event A items). As is the case for the pIAT, the aIAT is relationally more complex than traditional IATs. Most importantly, in the aIAT, participants respond to statements (i.e., combinations of items) rather than individual elements of a statement. Nevertheless, unlike the RRT, the aIAT does not require participants to relate all elements of the to-be-measured belief (e.g., “Event A is true”) because responses can be selected on the basis of the individual elements of that belief (e.g., “Event A” or “true”). Therefore, like all other variants of the IAT, the aIAT does not require participant to respond on the basis of whether a statement is to be considered as true or false.

The fact that the RRT is fundamentally different from both the pIAT and aIAT does not, however, allow for the conclusion that only the RRT can be used to capture beliefs at the implicit level. In fact, we believe that scores on the pIAT, aIAT, and even traditional IATs reflect beliefs (also see De Houwer, 2014). Although IATs do not require participants to relate all elements of a to-be-measured belief, participants might still relate those elements to one another, either explicitly or implicitly. Take the self-esteem IAT that we mentioned in the introduction. Rather than responding to self-related items merely on the basis of the fact that the item refers to oneself, participants could recode the task in such a way that they respond to self-related items as positive items (i.e., based on the fact that self-related items such as “I” have a positive valence for the participant). This would imply that self-related items are responded to in terms of how they relate to positive and negative valence, which could reflect beliefs about those items (e.g., “I like myself”). Importantly, the way in which items are responded to can vary in implicit, unintentional ways. For instance, participants might sometimes erroneously classify self-related items on the basis of their valence even when they do not have the intention to do so. In fact, one of the most successful accounts of the IAT implies that IAT effects are due to such instances of task misapplication (i.e., Klauer and Mierke, 2005). Hence, IAT effects might well reflect the implicit (in the sense of fast and unintentional) endorsement of propositions.

Because traditional IATs do not incorporate any information about the relation between the different categories, different participants might (implicitly or explicitly) relate the categories of the IAT in different ways. For instance, whereas non-dysphoric students might relate items that refer to the self and positive items in terms of liking (i.e., respond to items in terms of whether they refer to something you like), dysphoric students might relate those items in terms of aspirations (i.e., respond to items in terms of whether they are something you want). Although this idea leaves open the question of why dysphoric and non-dysphoric participants differ in this manner, it is in line with the fact that self-esteem IAT scores do not differ between those participants even though all other evidence leads to the conclusion that dysphoric participants do have lower self-esteem (De Raedt et al., 2006). It is also in line with the IRAP findings of Remue and colleagues (Remue et al., 2013, 2014) which suggest that dysphoric and non-dysphoric students do indeed differ in their implicit endorsement of the propositions “I am good” and “I want to be good.” From this perspective, pIATs and aIATs differ from traditional IATs in that the former are more likely to guide the way in which participants relate the different categories by specifying the precise way in which those stimuli should be related (e.g., “I like” or “I dislike”).

In short, it seems reasonable to assume that scores on IATs (and other implicit measures; see De Houwer, 2014) reflect beliefs at the implicit level. Still, the RRT is likely to have advantages over other implicit measures when it comes to capturing beliefs. Most importantly, because the RRT requires participants to relate the elements of beliefs in a highly specific manner, the RRT offers more control over the way that participants relate stimuli and thus over the type of beliefs that scores on the RRT capture. Of course, whether the RRT (or IRAP) outperforms the IAT as an implicit measure of beliefs is an empirical issue.

Finally, a reviewer alerted us to the fact that the RRT bears some resemblance to one particular variant of the IRAP in which participants are cued to lie or respond truthfully (Levin et al., 2010). When the word LIE is presented on the screen, participants select response “similar/yes” if they consider the two concepts on the screen to be dissimilar (e.g., “addict” and “good”) and to select the response “dissimilar/no” if they consider the concepts to be similar (e.g., “addict” and “bad”). On trials with the word TRUTH, “similar/yes” has to be selected for similar pairs and “dissimilar/no” for dissimilar pairs. Unlike to what is the case in the RRT, in this variant of the IRAP, the experimenter does not determine which responses are correct or incorrect. Instead, it is the participant who first has to decide what is true for him or her, after which he or she can select the response in line with the cue on that trial (TRUTH or LIE). Hence, this version of the IRAP seems to tap into how well people can intentionally lie about their beliefs (relative to telling the truth). In the RRT, on the other hand, participants are instructed by the experimenter to respond in a particular manner that might be either consistent or inconsistent with the beliefs of the participant. The task does not require participants to decide what their beliefs are. Which responses qualify as correct depends entirely on the experimenter. As such, RRT effects do not capture the ability to lie in an intentional manner (i.e., to retrieve and then conceal the truth) but the ability to act in ways that are consistent or inconsistent with a belief one might have. Although it needs to be settled empirically which of the two tasks is more valid and useful, this variant of the IRAP does differ from the RRT in non-trivial ways. Moreover, as is the case in most instantiations of the IRAP, attrition rates in this variant were very high (25%), probably because of the trial-by-trial changes in the location of the response options and the nature of response cue. Because we observed much a lower attrition rate in our RRT study, it does seem to be the case that the RRT is more user friendly than the variant of the IRAP that was introduced by Levin et al. (2010).

To conclude, the present paper introduces the RRT as a tool for obtaining an implicit measure of beliefs. We reported a first study in which the RRT was used to measure the extent to which Flemish participants hold the belief that Flemish people are more intelligent than immigrants. As expected, scores on the RRT correlated with ratings of how much participants explicitly endorse this belief. Also correlations with questionnaires of racism were observed. Although these findings provide initial evidence for the claim that the RRT provides a way to capture beliefs, more research is clearly needed, especially research in which RRT scores are related with real-life behavior. Indeed, it might well be that RRT effects allow one to predict unique variance in behavior that cannot be predicted on the basis of explicit measures of beliefs. Such an added value is likely to arise in situations where implicitly endorsed propositions differ from explicitly endorsed propositions (see Shidlovski et al., 2014). Future work will also need to substantiate the claim that RRT scores qualify as an *implicit* measure of beliefs and to compare the RRT with other potential implicit measures of beliefs. Moreover, it would be good to directly compare RRT measures with other measures in terms of validity, utility, and employability. By providing the first step in the validation process, however, the present paper highlights the potential of the RRT and sets the stage for such work.

### Conflict of interest statement

The authors declare that the research was conducted in the absence of any commercial or financial relationships that could be construed as a potential conflict of interest.

## Footnotes

^1^Research on implicit measures has been conducted both within the cognitive and the functional tradition in psychology (De Houwer, 2011; Hughes et al., 2011). The term “implicit belief” could also be used either at the mental or at the functional level of explanation. At the mental level, it refers to an automatically constructed or activated propositional representation, that is, an informational unit in memory that contains information about how concepts are related and that mediates the impact of the environment on behavior by means of automatically operating mental processes (see De Houwer, 2009, 2014). At the functional level, implicit beliefs can be defined as specific patterns of (arbitrarily applicable) relational responding, that is, behavior that is under the control of the relation between events. Given the assumption that (arbitrarily applicable) relational responding is mediated by propositional representations (Hughes et al., 2011), measures such as IRAP scores can be conceptualized both as indices of relational responding (when adopting a functional perspective) and as indices of propositional representations (when adopting a mental perspective).

^2^It is also possible to calculate an IRAP score for each trial type separately, which can be used an index for four different beliefs). In the foregoing example, these trial types would be: “I am good,” “I am bad,” “I am not good,” and “I am not bad”(see Barnes-Holmes et al., 2010).

^3^In many instantiations of the IRAP, participants are trained to respond in certain ways via error feedback rather than given explicit instructions about the stimulus-response assignments

^4^The proposition that an association exists can be endorsed but the association itself cannot.
