# Supplementary material for: The relational responding task: toward a new implicit measure of beliefs
Source: Front Psychol. 2015 Mar 24;6:319. doi: 10.3389/fpsyg.2015.00319 (PMC4371587; doi:10.3389/fpsyg.2015.00319)
Supplement: Supplementary file 1 [file DataSheet1.DOCX]

**Appendix 1. Target statements presented during the RRT (English translation between parentheses)**

-Vlamingen zijn intelligenter dan allochtonen. (Flemish people are more intelligent than immigrants)

-Vlamingen zijn slimmer dan allochtonen. (Flemish people are smarter than immigrants)

-Vlamingen zijn verstandiger dan allochtonen. (Flemish people are wiser than immigrants)

-Vlamingen zijn scherpzinniger dan allochtonen. (Flemish people are sharper than immigrants)

-Vlamingen zijn minder dom dan allochtonen. (Flemish people are less dumb than immigrants)

-Allochtonen zijn niet zo intelligent als Vlamingen. (Immigrants are not as intelligent as Flemish people)

-Allochtonen zijn dommer dan Vlamingen. (Immigrants are dumber than Flemish people)

-Allochtonen zijn minder verstandig dan Vlamingen. (Immigrants are less wise than Flemish people)

-Allochtonen zijn minder scherpzinnig dan Vlamingen. (Immigrants are less sharp than Flemish people)

-Allochtonen zijn achterlijker dan Vlamingen. (Immigrants are more backward than Flemish people)

-Vlamingen zijn niet zo intelligent als allochtonen. (Flemish people are not as intelligent as immigrants)

-Vlamingen zijn dommer dan allochtonen. (Flemish people are dumber than immigrants)

-Vlamingen zijn minder verstandig dan allochtonen. (Flemish people are less wise than immigrants)

-Vlamingen zijn minder scherpzinnig dan allochtonen. (Flemish people are less sharp than immigrants)

-Vlamingen zijn achterlijker dan allochtonen. (Flemish people are more backward than immigrants)

-Allochtonen zijn intelligenter dan Vlamingen. (Immigrants are more intelligent than Flemish people)

-Allochtonen zijn slimmer dan Vlamingen. (Immigrants are smarter than Flemish people)

-Allochtonen zijn verstandiger dan Vlamingen. (Immigrants are wiser than Flemish people)

-Allochtonen zijn scherpzinniger dan Vlamingen. (Immigrants are sharper than Flemish people)

-Allochtonen zijn minder dom dan Vlamingen. (Immigrants are less dumb than Flemish people)
